# The association of dyslipidemia and peripheral diabetic neuropathy: the influence of urea

**DOI:** 10.1186/1758-5996-7-S1-A30

**Published:** 2015-11-11

**Authors:** Patrícia Carvalho Machado Aguiar, Marcus Vinícius Della Coletta, Jean Jorge Silva de Souza

**Affiliations:** 1 UFAM, Manaus, Brazil

## Background

Peripheral neuropathy is a common complication of diabetes mellitus, usually manifesting as axonal sensitive-motor symmetric polyneuropathy. In the EURODIAB study, total cholesterol and triglycerides were associated to peripheral diabetic neuropathy (PDN).

## Aims

Evaluate the influence of cholesterol, triglycerides and statin use on a clinical score of peripheral neuropathy in patients with diabetes mellitus.

## Methodology

Ninety consecutive patients at a university hospital in Manaus, Amazonas were included in this study. Eighty-six (95.6%) had type 2 diabetes, 66.7% were female, mean age was 56.2±12.8 yrs.-old and mean time of diabetes diagnosis was 11.5±9.5 yrs. They were evaluated using Michigan Neuropathy Screening Instrument (MNSI) and the clinical component of the Michigan Diabetic Neuropathy Score (MDNS). This component evaluates vibratory, painful and tactile sensitivities. Subjects who scored 7 or more on this component of the MDNS were considered as having PDN (Feldman et al., 1994). MNSI and MDNS clinical component scores were analyzed according to lipid profile and statin use.

## Results

According to MDNS clinical component, 20 (22.2%) patients had PDN. These patients, compared to those patients who did not have PDN, had more time of diabetes diagnosis (16.2±11.3 vs. 10.2±8.6 yrs.), more stroke (15 vs. 3%), more insulin use (75.0 vs. 48.6%) and higher serum urea levels (44.4±17.6 vs. 36.9±16.8 mg/dL). There were no differences between patients with and without PDN in relation to age, retinopathy, nephropathy previous diagnosis, coronary heart disease, glycated hemoglobin, serum creatinine or statin use. No association was found between PDN and cholesterol (r=0.1297, p=NS) or triglyceride levels (r=0.0315, p=NS). But, a positive correlation was found between serum urea levels and MDNS (r=0.2957, p<0.01). However, when considering only the 65 (72.2%) patients with serum urea below 50 mg/dL, there was a positive correlation between total cholesterol and MDNS (r=0.2580, p<0.05) and between triglycerides and MDNS (r=0.2585, p<0.05).

## Conclusion

In patients who had serum urea below 50 mg/dL, total cholesterol and triglycerides correlated weakly but significantly to MDNS. Patients who have high cholesterol and triglycerides levels should be considered at higher risk for PDN.

